# Daly/Cost comparison in the management of peripheral arterial disease at 17 Belgian hospitals

**DOI:** 10.1186/s12913-023-10535-2

**Published:** 2024-01-19

**Authors:** Benoît Rondelet, Fabian Dehanne, Julie Van Den Bulcke, Dimitri Martins, Asmae Belhaj, Benoît Libert, Pol Leclercq, Magali Pirson

**Affiliations:** 1 Department of Cardio-Vascular, Thoracic Surgery and Lung Transplantation, CHU UCL Namur, UCLouvain, Avenue G. Therasse, 1, 5530 Yvoir, Belgium; 2 Chief Medical Officer Department, CHU UCL Namur, UCLouvain, Yvoir, Belgium; 3 Chief Executive Officer Department, CHU UCL Namur, UCLouvain, Yvoir, Belgium; 4 Health and Society Research Institute (IRSS) – UCLouvain, Louvain-La-Neuve, Belgium; 5 Health Economics, Hospital Management and Nursing Research Department, School of Public Health, Université Libre de Bruxelles, Brussels, Belgium

**Keywords:** Peripheral arterial disease, DALY, Disability-Adjusted Life Year, Cost, Atherosclerosis, Complication

## Abstract

**Objective:**

Peripheral arterial disease (PAD) is a manifestation of atherosclerosis that affects the lower extremities and afflicts more than 200 million people worldwide. Because of limited resources, the need to provide quality care associated with cost control is essential for health policies. Our study concerns an interhospital comparison among seventeen Belgian hospitals that integrates the weighting of quality indicators and the costs of care, from the hospital perspective, for a patient with this pathology in 2018.

**Methods:**

The disability-adjusted life years (DALYs) were calculated by adding the number of years of life lost due to premature death and the number of years of life lost due to disability for each in-hospital stay. The DALY impact was interpreted according to patient safety indicators. We compared the hospitals using the adjusted values ​​of costs and DALYs for their case mix index, obtained by relating the observed value to the predicted value obtained by linear regression.

**Results:**

We studied 2,437 patients and recorded a total of 560.1 DALYs in hospitals. The in-hospital cost average [standard deviation (SD)] was €8,673 (€10,893). Our model identified the hospitals whose observed values were higher than predicted; six needed to reduce the costs and impacts of DALYs, six needed to improve one of the two factors, and four seemed to have good results. The average cost (SD) for the worst performing hospitals amounted to €27,803 (€28,358).

**Conclusions:**

Studying the costs of treatment according to patient safety indicators permits us to evaluate the entire chain of care using a comparable unit of measurement.

## Introduction

Atherosclerosis is a form of arterial ageing involving atherosclerotic plaque formation in the artery wall. The plaques consist of a lipid deposit (cholesterol, triglycerides, or both), limestone and inflammatory and muscular cells surrounded by a fibrous covering. These atheromatous plaques grow in the artery wall, which thickens and becomes stenotic. Lesion progression and plaque rupture can lead to thrombosis and arterial obstruction. Atherosclerosis mainly affects medium- and large-calibre arteries: the coronary arteries that irrigate the heart, the carotid arteries intended for the brain, the infrarenal abdominal aorta, the iliac arteries, and the femoral and femoro-popliteal arteries of the legs [1]. Peripheral arterial disease (PAD) is a manifestation of atherosclerosis that affects the lower limbs and is a major public health problem because it affects more than 200 million people worldwide [2].

According to the World Health Organization, cardiovascular disease, in general, is responsible for more than 30% of mortality, constituting the leading cause of death in the world [3]. Disability and mortality associated with PAD have increased over the past 20 years. Moreover, the burden of PAD is no longer limited to the elderly population but now affects young adults [4]. In France, the condition is responsible for an additional cost of more than €11,000 per year/patient. In comparison, the annual cost of a patient with a myocardial infarction amounts to €12,679 in the following year. Based on 500,000 French patients in 2014 insured at 100%, the annual cost of this pathology was estimated at €5,500 million [5]; the situation is proportionally comparable in Belgium.

Faced with this situation and due to financial resource limitations, the need to provide quality care associated with cost control is becoming important in health policies. A recent study using administrative data highlighted the crucial role of complications and readmissions after vascular surgery both as a measure of quality and as a major source of cost savings for healthcare systems [6]. A simultaneous analysis of costs and results therefore appears useful to reflect the areas for improving healthcare by working on good practice guides [7, 8] coupled with interhospital benchmarking.

The disability-adjusted life year (DALY) is a measurement unit now commonly used in health economics studies to reflect treatment or intervention effectiveness; it is also used as a medico-economic factor on a scale of 0 (perfect health) to 1 (equivalent to death) that indicates the burden of a disease. In 2015, Belgium recorded more than 100 years of life lost (YLLs) in good health, including harm to patients, per 100,000 inhabitants. The DALY average for the Organization for Economic Cooperation and Development (OECD) is more than 70 DALYs/100,000 inhabitants [9].

Our study proposes an interhospital comparison among 17 Belgian hospitals that integrates the weighting of quality indicators (DALY) and the costs of complete care for a patient from the Associated Hospital Cost Analysis Project (PACHA) [10].

## Methods

We used the methodology already reported by Dehanne et al. [11]. For the reader’s understanding, we have taken it up in extenso in the following paragraphs.

### Case selection

The study sample was based on data from 17 general hospitals in Belgium (including academic hospitals) from the Associated Hospital Cost Analysis Project (PACHA, Table 1). The hospitals involved have been anonymized. Institutional Review Board approval was not required for that study. We focused our analysis on inpatients with a primary diagnosis of PAD. Codes from I70.2* to I70.9* according to the 10th Revision of the International Statistical Classification of Diseases and Related Health Problems (ICD-10-BE) [12] were used to select these in-hospital stays. Within the diagnosis-related group (DRG), the admissions were then classified into three categories according to the Healthcare Cost and Utilization Project (HCUP) classification [13]: atherosclerosis (lower limbs), graft/prothesis complications, and gangrene. The minimum of one-night hospital stays in 2018 were kept for the study. Among these, we identified all patients who were readmitted to the same hospital within 30 days of discharge from the first inpatient stay. The inpatient stay in our study is therefore defined as the combination of the first in-hospital stay and the readmission, if it existed, of the same patient to the same hospital for a problem related to the first admission. Ultimately, our total population included 2,437 hospital stays. Among all these stays and according to group 34, we identified 19 different DRGs with diagnosis of atherosclerosis in the lower limbs.

**Table 1:** List of hospitals in the Associated Hospital Cost Analysis Project (PACHA)

Hospitals	Status	n of Beds
Centre Hospitalier de l’Ardenne	General	96
Centre Hospitalier Régional de Huy	General	317
Centre Hospitalier Régional de Namur	General	397
Centre Hospitalier Régional du Val de Sambre	General	297
Centre Hospitalier Universitaire de Tivoli	General	518
Centre Hospitalier Universitaire Saint-Pierre	General	441
Centre Hospitalier Interrégional Edith Cavell—Delta	General	438
Centre Hospitalier Régional Verviers	General	441
CHU UCL Namur—Godinne	Academic	386
CHU UCL Namur—Sainte-Elisabeth	General	305
Clinique St-Luc Bouge	General	302
Cliniques du Sud Luxembourg	General	337
Cliniques Universitaires Saint-Luc	Academic	973
Hôpital André Vésale	General	351
Hôpital Civil Marie Curie	General	547
Hôpital Erasme	Academic	884
Hôpitaux de Marche-en-Famenne et de Bastogne	General	199

### Patient safety indicators and Charlson index

To develop the patient safety indicators, we used the construction methodology of the Agency for Healthcare Research and Quality (AHRQ), V.5.0.8 [14]. The AHRQ’s indicators are measures of healthcare quality based on medico-administrative data available in hospital databases. Only the secondary diagnostic codes mentioned as «not present at admission» were used to identify hospital stay-related complications (Table 2). The Charlson index [15] was applied to the entire population.

**Table 2:** List of Patient safety indicators, AHRQ

List of patient safety indicators (PSI) from AHRQ V.5.0 used in the study	
PSI 03	Pressure ulcer rate
PSI 06	Iatrogenic pneumothorax rate
PSI 07	Central venous catheter-related bloodstream infection rate
PSI 09	Postoperative bleeding rate or hematoma rate
PSI 10	Postoperative physiological and metabolic disorder rate
PSI 11	Postoperative respiratory failure rate
PSI 12	Deep vein thrombosis rate or postoperative pulmonary embolism
PSI 13	Postoperative sepsis rate
PSI 16	Number of transfusion reactions

Belgian hospitals depend on a tool from 3 M^©^ to determine the severity of stays. This classification depends on co-morbidities, secondary problems and procedures carried out during the stay. This medico-economic classification relates the level of resources that hospitals must provide to care the patient.

Here, we have used the Charlson index to avoid the over-coding bias associated with severity indices. The Charlson score is calculated using the medical information available at the beginning of the stay.

The HCUP classification was then used to avoid heterogeneity within certain disease groups. Given the origin of these codes, we believe that they are closely linked to our cost evaluation objectives. By using HCUP codes, we go from 86 different ICD10 codes (main diagnosis) to 3 HCUP disease groups. This conversion seems easier to use for statistical purposes.

### Calculation of DALYs

DALYs were calculated by adding the number of years of life lost due to premature death [years of life lost (YLLs)] and the number of years of life lost due to disability [years of life lost due to disability] (YLDs)] [16] for each hospital stay.

$${\varvec{D}}{\varvec{A}}{\varvec{L}}{\varvec{Y}}{\varvec{s}}={\varvec{Y}}{\varvec{L}}{\varvec{L}}{\varvec{s}}+{\varvec{Y}}{\varvec{L}}{\varvec{D}}{\varvec{s}}$$

Specifically, the number of YLDs was calculated by multiplying incident cases by the duration and severity of disability for a given disease. For pressure ulcers (stage III and IV) and postoperative respiratory failure, we used the disability weight from the 2016 Global Burden of Disease (Institute for Health Metrics and Evaluation report) [17], while for the other complications, the weights from the article by Jha and colleagues [18] were applied.

When we were unable to determine the DALYs, we referred to a pathology that was clinically similar to our complication (e.g., respiratory failure or other severe cardiovascular diseases). The durations of short-term complications were taken from the literature review of Jha’s article [18]. The DALY calculation was then applied to all the stays of our population.

$$\boldsymbol N\boldsymbol u\boldsymbol m\boldsymbol b\boldsymbol e\boldsymbol r\boldsymbol\;\boldsymbol o\boldsymbol f\boldsymbol\;\boldsymbol y\boldsymbol e\boldsymbol a\boldsymbol r\boldsymbol s\boldsymbol\;\boldsymbol o\boldsymbol f\boldsymbol\;\boldsymbol l\boldsymbol i\boldsymbol f\boldsymbol e\boldsymbol\;\boldsymbol l\boldsymbol o\boldsymbol s\boldsymbol t\boldsymbol\;\boldsymbol d\boldsymbol u\boldsymbol e\boldsymbol\;\boldsymbol t\boldsymbol o\boldsymbol\;\boldsymbol d\boldsymbol i\boldsymbol s\boldsymbol a\boldsymbol b\boldsymbol i\boldsymbol l\boldsymbol i\boldsymbol t\boldsymbol y\boldsymbol\;\boldsymbol(\boldsymbol P\boldsymbol a\boldsymbol t\boldsymbol i\boldsymbol e\boldsymbol n\boldsymbol t\boldsymbol\;\boldsymbol S\boldsymbol a\boldsymbol f\boldsymbol e\boldsymbol t\boldsymbol y\boldsymbol)\boldsymbol\;\boldsymbol p\boldsymbol e\boldsymbol r\boldsymbol\;\boldsymbol h\boldsymbol o\boldsymbol s\boldsymbol p\boldsymbol i\boldsymbol t\boldsymbol a\boldsymbol l\boldsymbol\;\boldsymbol s\boldsymbol t\boldsymbol a\boldsymbol y\boldsymbol\;\boldsymbol(\boldsymbol Y\boldsymbol L\boldsymbol D\boldsymbol s\boldsymbol)\boldsymbol=\boldsymbol W\boldsymbol e\boldsymbol i\boldsymbol g\boldsymbol h\boldsymbol t\boldsymbol\;\boldsymbol o\boldsymbol f\boldsymbol\;\boldsymbol t\boldsymbol h\boldsymbol e\boldsymbol\;\boldsymbol c\boldsymbol o\boldsymbol m\boldsymbol p\boldsymbol l\boldsymbol i\boldsymbol c\boldsymbol a\boldsymbol t\boldsymbol i\boldsymbol o\boldsymbol n\boldsymbol\;\boldsymbol X\boldsymbol\;\boldsymbol D\boldsymbol u\boldsymbol r\boldsymbol a\boldsymbol t\boldsymbol i\boldsymbol o\boldsymbol n\boldsymbol\;\boldsymbol o\boldsymbol f\boldsymbol\;\boldsymbol t\boldsymbol h\boldsymbol e\boldsymbol\;\boldsymbol c\boldsymbol o\boldsymbol m\boldsymbol p\boldsymbol l\boldsymbol i\boldsymbol c\boldsymbol a\boldsymbol t\boldsymbol i\boldsymbol o\boldsymbol n$$

We also assigned a DALY for stays for which readmission for pain/discomfort occurred within 30 days and which was related to the initial reason for hospitalization. The duration of disability for readmissions corresponded to the sum of the duration of the first stay and the period preceding the start of the second admission. Mortality was calculated based on Belgian mortality and life expectancy tables [19]. The disability weighting of death corresponding to 1 in our study was multiplied by life expectancy according to the individual’s age.

$$\boldsymbol N\boldsymbol u\boldsymbol m\boldsymbol b\boldsymbol e\boldsymbol r\boldsymbol\;\boldsymbol o\boldsymbol f\boldsymbol\;\boldsymbol y\boldsymbol e\boldsymbol a\boldsymbol r\boldsymbol s\boldsymbol\;\boldsymbol o\boldsymbol f\boldsymbol\;\boldsymbol l\boldsymbol i\boldsymbol f\boldsymbol e\boldsymbol\;\boldsymbol l\boldsymbol o\boldsymbol s\boldsymbol t\boldsymbol\;\boldsymbol d\boldsymbol u\boldsymbol e\boldsymbol\;\boldsymbol t\boldsymbol o\boldsymbol\;\boldsymbol d\boldsymbol e\boldsymbol a\boldsymbol t\boldsymbol h\boldsymbol\;\boldsymbol p\boldsymbol e\boldsymbol r\boldsymbol\;\boldsymbol h\boldsymbol o\boldsymbol s\boldsymbol p\boldsymbol i\boldsymbol t\boldsymbol a\boldsymbol l\boldsymbol\;\boldsymbol s\boldsymbol t\boldsymbol a\boldsymbol y\boldsymbol\;\boldsymbol(\boldsymbol Y\boldsymbol L\boldsymbol L\boldsymbol s\boldsymbol)\boldsymbol=\mathbf1\boldsymbol\;\boldsymbol X\boldsymbol\;\boldsymbol N\boldsymbol u\boldsymbol m\boldsymbol b\boldsymbol e\boldsymbol r\boldsymbol\;\boldsymbol o\boldsymbol f\boldsymbol\;\boldsymbol y\boldsymbol e\boldsymbol a\boldsymbol r\boldsymbol s\boldsymbol\;\boldsymbol o\boldsymbol f\boldsymbol\;\boldsymbol l\boldsymbol i\boldsymbol f\boldsymbol e\boldsymbol\;\boldsymbol l\boldsymbol o\boldsymbol s\boldsymbol t\boldsymbol/\boldsymbol a\boldsymbol g\boldsymbol e$$

If a patient experienced a complication followed by a death during their stay, we only counted this case as a death.

### Hospital cost data

In this study, costs refer to expenses for the acute management of hospital stays from the hospital perspective. The cost from the hospital perspective was calculated using an analytical accounting method for full costing [10]. As not all hospitals have a revalidation department, we did not consider cost data related to activities that occurred in the revalidation department to objectively compare them. The isolated costs of revalidation have then been subtracted from the total cost of the stay.

$$\boldsymbol T\boldsymbol o\boldsymbol t\boldsymbol a\boldsymbol l\boldsymbol\;\boldsymbol c\boldsymbol o\boldsymbol s\boldsymbol t\boldsymbol\;\boldsymbol o\boldsymbol f\boldsymbol\;\boldsymbol i\boldsymbol n\boldsymbol p\boldsymbol a\boldsymbol t\boldsymbol i\boldsymbol e\boldsymbol n\boldsymbol t\boldsymbol=\boldsymbol(\boldsymbol t\boldsymbol h\boldsymbol e\boldsymbol\;\boldsymbol c\boldsymbol o\boldsymbol s\boldsymbol t\boldsymbol\;\boldsymbol o\boldsymbol f\boldsymbol\;\boldsymbol t\boldsymbol h\boldsymbol e\boldsymbol\;\boldsymbol f\boldsymbol i\boldsymbol r\boldsymbol s\boldsymbol t\boldsymbol\;\boldsymbol a\boldsymbol d\boldsymbol m\boldsymbol i\boldsymbol s\boldsymbol s\boldsymbol i\boldsymbol o\boldsymbol n\boldsymbol-\boldsymbol t\boldsymbol h\boldsymbol e\boldsymbol\;\boldsymbol c\boldsymbol o\boldsymbol s\boldsymbol t\boldsymbol\;\boldsymbol r\boldsymbol e\boldsymbol l\boldsymbol a\boldsymbol t\boldsymbol e\boldsymbol d\boldsymbol\;\boldsymbol t\boldsymbol o\boldsymbol\;\boldsymbol t\boldsymbol h\boldsymbol e\boldsymbol\;\boldsymbol r\boldsymbol e\boldsymbol v\boldsymbol a\boldsymbol l\boldsymbol i\boldsymbol d\boldsymbol a\boldsymbol t\boldsymbol i\boldsymbol o\boldsymbol n\boldsymbol)\boldsymbol\;\boldsymbol+\boldsymbol\;\boldsymbol t\boldsymbol h\boldsymbol e\boldsymbol\;\boldsymbol t\boldsymbol o\boldsymbol t\boldsymbol a\boldsymbol l\boldsymbol\;\boldsymbol o\boldsymbol f\boldsymbol\;\boldsymbol t\boldsymbol h\boldsymbol e\boldsymbol\;\boldsymbol r\boldsymbol e\boldsymbol a\boldsymbol d\boldsymbol m\boldsymbol i\boldsymbol s\boldsymbol s\boldsymbol i\boldsymbol o\boldsymbol n$$

### Statistical analysis

Statistical analyses were performed using SPSS software, V.25. The descriptive statistics of the different variables are presented as the mean (SD).

Kruskal‒Wallis and Mann‒Whitney tests were used to verify the significant differences in dependent variables (DALY/cost) in relation to ordinal and dichotomous independent variables.

The indicators structured according to the Donabedian model were constructed based on data from the literature and the availability of data in our database [20–23].

To correct the distribution of dependent variables such as DALY and cost, a logarithmic transformation was used. We then recoded our independent variables into a dummy variable. A stepwise linear regression was then performed on these new dependent variables to identify predicted hospital values. This approach allowed data to be adjusted according to the seriousness of the pathologies specific to each healthcare establishment.

The predictive indices used in our model were the Charlson comorbidity index, age, admission diagnosis, sex, type of admission, type of destination on discharge, passage to the intensive care unit, transfer to the geriatric unit, geriatric assessment during hospitalization, readmission within 30 days of the end of the first stay, and patient safety indicators during the hospital stay. We selected these independent variables based on indicators taken from the literature [20–23] and on the significance of the data from the univariate analysis. Homoscedasticity was checked using a graph.

Preference was given to using the Charlson index in the regression, rather than the relative weight (case mix index), since the Charlson index includes comorbidities present at admission and not complications encountered during the hospital stay. Finally, ratios between the observed value and predicted value of the inpatient stay were calculated.

## Results

Table 3 describes the main results from the univariate analysis and hospital comparison. Table 3 summarizes the main regression results.

**Table 3:** Distribution of HCUP-severity

*n* = 2400		Atherosclerosis	Graft/Prosthetic complications	Gangrene
1-Minor	n	925	34	16
	%	45%	32%	7%
2-Moderate	n	913	54	106
	%	44%	51%	46%
3-Major	n	193	15	84
	%	9%	14%	36%
4-Extreme	n	32	2	26
	%	2%	2%	11%
**Total**		**2063**	**105**	**232**

### Description of inpatients

#### Population

We studied 2,437 inpatients for the three HCUP codes: atherosclerosis (*n* = 2,097), prosthetic complications (*n* = 107), and gangrene (*n* = 233) (Table 3). The population on average (SD) was 68 years (11 years) in age, with 67% being male. Most patients (59%) were in their fifties. The Charlson index average (SD) calculated was 2.7 (1.7) with a distribution of 24%, 31%, 20%, 11% and 13% for the indices from 1 to 5, respectively. Eighty-four percent of the population had a severity index of -1 (minor; 40%) or -2 (moderate; 44%) (Table 4).

**Table 4:** Description of the study population

**Dimensions**	**n**	**%**	**DALYs**			**Cost (€)**		
			Mean	SD	p	Mean	SD	p
**Hospital Stays**	2,437	100.0	0.23	1.79		8,673	10,893	
**HCUP code**								
Atherosclerosis	2,097	86.0	0.13	1.34	0.000 *	7,713	9,032	0.000 *
Graft/prosthetic complications	107	4.4	0.21	1.51		10,417	14,247	
Gangrene	233	9.6	1.10	3.92		16,693	18,118	
**Gender**								
Male	1,630	66.9	0.24	1.78	0.900 **	8,646	10,700	0.938 **
Female	807	33.1	0.20	1.80		8,780	11,104	
**Age category**								
1 - (25-49)	94	3.9	0.01	0.01	0.157 *	8,517	6,909	0.271 *
2 - (50-59)	430	59.3	0.08	1.60		8,409	11,329	
3 - (60-69)	819	29.9	0.29	2.24		8,537	11,314	
4 - (70-79)	684	7.4	0.19	1.50		8,568	9,805	
5 - (80-99)	410	1.8	0.40	1.57		9,537	11,665	
**Severity**								
1 – Minor	975	40.0	0.00	0.00	0.000 *	5,782	5,380	0.000 *
2 - Moderate	1,073	44.0	0.11	1.15		8,023	7,043	
3 - Major	292	12.0	0.46	2.20		15,276	15,072	
4 - Extreme	60	2.5	4.98	7.70		35,155	35,359	
**Mortality**								
1 – Minor	1,446	59.3	0.01	0.44	0.000 *	6,476	6,313	0.000 *
2 - Moderate	728	29.9	0.14	1.23		9,732	9,622	
3 – Major	181	7.4	0.94	3.54		14,984	16,504	
4 - Extreme	45	1.8	5.89	7.55		36,773	36,984	
**Charlson index**								
1	583	24.0	0.45	0.76	0.000 *	6,131	8,124	0.000 *
2	767	31.0	0.16	1.48		7,730	8,306	
3	495	20.0	0.27	1.96		9,652	12,007	
4	265	11.0	0.26	1.30		10,112	10,670	
5	317	13.0	0.63	3.22		13,113	13,000	
**Admission type**								
Planned	2,211	90.7	0.15	1.41	0.000 *	7,900	9,649	0.000 *
Emergency via ED	206	8.5	1.09	3.96		16,621	17,251	
Emergency without ED	20	0.8	0.17	0.72		14,461	14,735	
**Patient origin**								
Residence	2,371	59.3	0.22	1.79	0.259 *	8,541	10,499	0.019 *
Other hospital	27	29.9	0.69	2.53		14,257	17,445	
Care home	33	7.4	0.34	1.10		11,825	11,097	
Public place	6	1.8	0.01	0.01		25,393	44,545	
**Patient addressing**								
Own initiative	87	3.6	1.17	4.57	0.000 *	14,345	16,415	0.000 *
General practitioner	89	3.7	0.85	3.24		17,061	15,295	
Consultant	2,232	91.6	0.17	1.48		8,081	10,136	
Third	29	1.2	0.14	0.70		12,987	9,557	
**Destination**								
Residence	2,270	93.1	0.00	0.01	0.000 *	7,992	9,268	0.000 *
Other hospital	43	1.8	0.02	0.06		22,500	29,208	
Care home	70	2.9	0.01	0.04		13,628	13,250	
Deceased	52	2.1	10.53	6.46		20,112	21,082	
Others	2	0.1	0.00	0.00		35,221	34,718	
**Care type**								
Medical	339	13.9	0.42	2.27	0.007 **	7,689	10,312	0.000 **
Surgery	2,098	86.1	0.20	1.69		8,852	10,909	
**Intensive care unit**								
No	2,225	91.3	0.12	1.15	0.000 **	7,440	7,790	0.000 **
Yes	212	8.7	1.37	4.63		21,818	22,927	
**Geriatric care unit**								
No	2,383	97.8	0.21	1.75	0.083 **	8,571	8,691	0.000 **
Yes	54	2.2	1.15	2.69		2,383	2,437	
**Inpatient geriatric liaison**								
No	2,346	96.3	0.21	1.77	0.004 **	8,502	10,744	0.000 **
Yes	91	3.7	0.61	2.13		13,551	12,008	
**Readmission <30 days**								
0	2,084	85.5	0.27	1.93	0.000 *	7,257	8,409	0.000 *
1	328	13.5	0.01	0.02		17,641	17,973	
2	25	1.0	0.01	0.00		10,748	9,814	
**Outcome Complications**	582	23.9	0.96	3.56	0.000 **	14,075	16,802	0.000 **
**Pressure ulcer (III or IV)**								
No	2,433	99.8	0.23	1.79	0.000 **	8,610	10,222	0.006 **
Yes	4	0.2	0.32	0.05		57,906	85,813	
**Iatrogenic pneumothorax**								
No	2,437	100.0	0.23	1.79	NA	8,691	10,834	NA
Yes	0	0.0	0.00	0.00		0	0	
**Catheter infection**								
No	2,372	97.3	0.19	1.67	0.000 **	8,123	8,783	0.000 **
Yes	65	2.7	1.66	4.05		29,401	34,089	
**Perioperative haemorrhage or haematoma**								
No	1,955	80.2	0.17	1.52	0.000 **	7,526	8,772	0.000 **
Yes	482	19.8	0.47	2.59		13,414	15,935	
**Postoperative physiologic and metabolic derangement**								
No	2,375	97.5	0.16	1.38	0.000 **	8,222	9,125	0.000 **
Yes	62	2.5	2.95	6.77		26,646	33,320	
**Postoperative respiratory failure**								
No	2,417	99.2	0.15	1.31	0.000 **	8,339	9,463	0.000 **
Yes	20	0.8	10.38	9.00		51,199	41,742	
**Perioperative pulmonary embolism or deep venous thrombosis**								
No	2,433	99.8	0.22	1.76	0.000 **	8,667	10,809	0.024 **
Yes	4	0.2	4.17	8.16		23,272	17,311	
**Sepsis**								
No	2,424	99.5	0.21	1.71	0.000 **	8,504	10,081	0.000 **
Yes	13	0.5	3.93	6.32		43,499	44,548	
**Transfusion reaction**								
No	2,437	100.0	0.23	1.79	NA	8,691	10,834	NA
Yes	0	0.0	0.00	0.00		0	0	
**Death**								
No	2,385	97.9	0.00	0.02	0.000 **	8,442	10,369	0.000 **
Yes	52	2.1	10.53	6.46		20,112	21,082	

*HCUP* Healthcare Cost and Utilization Project, *ED* Emergency department, *DALYs* Disability-Adjusted Life Years, *DS* Deviation standard

* Kruskal‒Wallis test

** Mann‒Whitney test

The patients were admitted to the 17 hospitals according to an urgent mode with (*n* = 206; 9%) or without (*n* = 20) visiting the emergency department, or according to a planned mode (*n* = 2,211; 91%). The mean length (SD) of stay was 6.6 days (11.4 days) (Table 4). These stays included 9% admissions to intensive care and 2% hospitalizations in the geriatrics department. During their stay, 4% of patients benefited from contact with a physician specializing in geriatrics (Table 4). The average length of stay (SD) for all stays was 6.53 days (1.36 days).

The mortality was 2.1%, and the complication rate during hospital stays was 23.9%, divided into bedsores (< 1%), catheter-related bloodstream infections (3%), haemorrhages and haematomas (20%), physiological and/or metabolic disorders (3%), respiratory failure (1%), deep vein thrombosis or pulmonary embolism (< 1%), and sepsis (1%). There were no reports of iatrogenic pneumothorax or transfusion reactions in the database in 2018 (Table 4). Readmissions accounted for 13% of stays, and 1% of patients had been admitted twice in the first 30 days following the initial discharge (Table 4).

#### Duration and cost of stay

The average cost (SD) of a hospital stay was €8,673 (€10,893) or higher (*p* < 0.001) for surgical treatment (€8,852 ± €10,909) vs. medical treatment (€7,689 ± €10,312) (Table 4).

This cost was significantly different (*p* < 0.001) according to the reported HCUP code [mean (M) ± SD]: atherosclerosis (€7,713 ± €9,032), graft/prosthetic complications (€10,417 ± €14,247), and gangrene (€16,693 € ± €1,118) (Table 4).

Age and sex did not modify the costs of care (Table 4).

The average hospitalization cost (SD) was significantly modified by the severity index (*p* < 0.001) and the Charlson index (*p* < 0.001). The average cost (SD) went, respectively from €5,782 (€5,380) to €35,155 (€35,359) for an admission of severity 1-minor or severity 4-extreme and from €6,131 (€8,124) to €13,113 (€13,000) for patients scored as category -1 and -5 on the Charlson index (*p* < 0.001) (Table 4).

We also measured changes in average costs (SD) (*p* < 0.001) for patients with a 1-minor mortality index with €6,476 (€6,313) to €36,773 (€36,984) with an index of 4-extreme mortality (Table 4).

The total cost (SD) average for inpatients admitted for scheduled care was €7,900 (€9,649), while patients admitted urgently with or without visiting the emergency department were significantly higher (*p* < 0.001): €16,621 (€17,251) vs. €14,461 (€14,735), respectively (Table 4).

As illustrated in Table 3, patients referred by a care home had the lowest average cost (SD): €8,081 ± €10,136 (*p* < 0.05).

Patients discharged from hospital to home had an average cost (SD) of €7,992 (€9,268), while those transferred to another hospital or to a care home had an average cost of €22,500 (€29,208) or €13,628 (€13,250), respectively. If they died, the average cost (SD) of the stay was €20,112 (€21,082) (*p* < 0.001) (Table 4).

The average cost (SD) of readmission was estimated at €17,641 (€17,973) and was significantly higher than that of hospitalization without complications (€7,257 ± €8,429) (*p* < 0.001) (Table 3).

The average cost (SD) of a hospital stays requiring intensive care was €21,818 (€22,927), which was statistically higher (*p* < 0.001) than that which did not require ICU treatment (€7,440 ± €7,790) (Table 4).

Admitting a geriatric patient to the ad hoc unit costed €2,383 ± €2,437 on average (SD), which was lower than the average cost (SD) of conventional hospitalization (€8,571 ± €8,691) (*p* < 0.001). In comparison, the average cost (SD) of hospitalization in the vascular surgery department with geriatric contact (without completion under geriatric care) was significantly higher (€13,551 ± €12,008) (Table 4).

#### Cost associated with complications

In total, 582 of the 2,437 stays for PAD presented a complication. The hospital stay mean cost (SD) for a complication was €14,075 (€16,802), which was approximately 62% higher than that of in-hospital care without complications (*p* < 0.001) (Table 4). The average costs of hospitalizations related to complications were significantly increased for the treatment of pressure ulcers (€57,906 ± €85,813 vs. €8,610 ± €10,222; *p* < 0.01), catheter-related bloodstream infections (€29,401 ± €34,089 vs. €8,128 ± €8,783; *p* < 0.001), bleeding and haematomas (€13.414 ± €15.935 vs. €7.526 ± €8.772; *p* < 0.001), physiological and/or metabolic disorders (€26.646 ± €33.320 vs. €8.222 ± €9.125; *p* < 0.001), respiratory failure (€51,199 ± €41,742 vs. €8,339 ± €9,463; *p* < 0.001), deep vein thrombosis or pulmonary embolism (€23,272 ± €17,311 vs. €8,667 ± €10,809; *p* < 0.05), and sepsis (€43.499 ± €44.548 vs. €8.504 ± €10.081; *p* < 0.001) (Table 4).

#### Impact of DALYs

We registered a total of 560.51 DALYs for these inpatients in the 17 hospitals in our study (Table 4). Deaths alone accounted for over 547.6 DALYs (YLLs) (Table 5). The mean number (SD) of DALYs per hospitalized patient was 0.23 (1.79) (Table 4). The mean number of DALYs was significantly different between the three HCUP codes: atherosclerosis (0.13 ± 1.34), prosthetic complications (0.21 ± 1.51), and gangrene (1.10 ± 3.92) (Table 4). Neither age nor sex influenced the average DALY calculation in our study (Table 4).

**Table 5:** Comparison of Donabedian indicators for the management of peripheral arterial disease in 17 Belgian hospitals

	**Hospitals**		**1**	**2**	**3**	**4**	**5**	**6**	**7**	**8**	**9**	**10**	**11**	**12**	**13**	**14**	**15**	**16**	**17**	*p*
**HCUP code**	Atherosclerosis (n)		233	13	93	63	175	288	178	115	231	83	72	133	81	89	43	146	61	
	Graft/Prosthetic complications (n)		11		4	4	2	21	5	17	12	12	2	1		1	3		12	
	Gangrene (n)		49	10	5	4	18	27	23	6	16	23	27	4	4	10	2	5		
	Total (n)		293	23	102	71	195	336	206	138	259	118	101	138	85	100	48	151	73	
**Population**	Gender (%)	Male	64	64	64	52	68	70	72	66	69	58	67	67	73	64	65	55	75	
		Female	36	36	36	48	32	30	28	34	31	42	33	33	27	36	35	45	25	
	Age (year)	M	67.2	74.1	64.2	71.2	67.5	70.9	66.4	69.4	68.9	68.7	67.4	67.8	68.9	72.5	66.9	68.5	66.9	
		SD	11.6	10.9	10.8	9.2	11.5	10.7	9.9	11.8	10.4	9.1	10.5	11.4	11.4	12.1	11.9	9.8	9.8	
	Charlson index	M	3.1	3.5	2.2	2.0	2.6	3.1	2.9	2.6	2.5	2.9	3.3	2.5	2.1	2.3	2.8	2.5	2.2	
		D	1.7	1.9	1.4	1.2	1.6	2.0	1.9	1.6	1.5	1.8	2.1	1.2	1.0	1.4	1.7	1.8	1.1	
**Stay**	Admission type	Planned	267	17	93	67	176	295	184	125	249	102	89	129	80	91	37	146	64	
		Emergency via ED	25	6	9	4	16	33	20	13	8	16	12	9	5	7	11	4	8	
		Emergency without ED	1				3	8	2		2					2		1	1	
	Length of stay	M	8.02	17.91	3.70	4.32	7.72	5.80	7.60	4.89	6.58	8.40	7.34	4.05	5.44	7.83	8.57	5.56	3.89	
		SD	9.00	23.75	3.89	6.58	14.09	10.83	17.22	6.68	12.28	14.23	10.03	5.96	9.47	9.59	7.31	12.78	4.64	
	Intensive care unit (%)		10.9	0.0	6.9	4.2	4.1	0.3	17.5	3.6	14.3	11.9	8.9	4.3	8.2	29.0	22.9	2.6	4.1	
	Geriatric care unit (%)		0.7	17.4	1.0	2.8	4.6	0.6	1.5	4.4	0.4	0.9	1.0	2.9	7.1	4.0	4.2	3.3	1.4	
	Inpatient geriatric liaison (%)		6.8	0.0	5.9	0.0	17.4	1.5	4.9	1.4	0.0	4.2	2.0	0.7	0.0	3.0	0.0	2.0	0.0	
**Outcome complications**	Pressure ulcer (III or IV) (%)		0.2	0.0	0.0	0.0	0.0	0.0	0.0	0.0	0.5	0.0	0.0	0.0	0.0	0.0	0.0	0.0	0.0	
	Iatrogenic pneumothorax (%)		0.0	0.0	0.0	0.0	0.0	0.0	0.0	0.0	0.0	0.0	0.0	0.0	0.0	0.0	0.0	0.0	0.0	
	Catheter infection (%)		2.3	0.2	0.0	0.2	0.3	0.8	0.6	0.2	1.4	1.1	0.3	0.5	0.3	0.6	0.9	0.5	0.0	
	Perioperative haemorrhage or haematoma (%)		4.9	0.0	2.2	1.2	8.2	8.5	5.8	5.8	12.6	5.5	0.2	2.8	3.2	5.8	1.8	3.4	2.2	
	Postoperative physiologic and metabolic derangement (%)		2.5	0.5	0.0	0.2	0.5	0.6	0.5	0.3	0.8	1.7	0.3	0.0	0.3	0.3	0.0	1.2	0.0	
	Postoperative respiratory failure (%)		0.0	0.0	0.0	0.2	0.5	0.2	0.5	0.2	0.3	0.3	0.2	0.0	0.3	0.2	0.5	0.0	0.0	
	Perioperative pulmonary embolism or deep venous thrombosis (%)		0.3	0.0	0.0	0.0	0.0	0.0	0.2	0.0	0.0	0.0	0.0	0.0	0.0	0.2	0.0	0.0	0.0	
	Sepsis (%)		0.0	0.2	0.0	0.2	0.3	0.0	0.2	0.0	0.5	0.2	0.2	0.0	0.2	0.2	0.2	0.0	0.0	
	Transfusion reaction (%)		0.0	0.0	0.0	0.0	0.0	0.0	0.0	0.0	0.0	0.0	0.0	0.0	0.0	0.0	0.0	0.0	0.0	
	Complications (%)		18.4	26.1	13.7	11.3	29.2	18.8	20.9	30.4	34.4	41.5	8.9	14.5	28.2	43.0	39.6	18.5	19.2	
**Postoperative evolution**	Case mix index *	M	2.04	1.08	1.69	1.69	2.04	1.79	1.64	1.82	1.85	1.81	1.87	1.81	1.90	1.75	2.25	1.83	2.02	0.0000
		SD	0.91	0.69	0.49	0.80	1.08	0.73	1.17	1.18	0.87	0.76	1.04	0.51	1.13	0.66	1.66	0.63	0.82	
	Mortality (%)		0.08	0.08	0.00	0.04	0.16	0.08	0.16	0.16	0.08	0.33	0.21	0.00	0.25	0.16	0.16	0.16	0.00	
	Readmission < 30 days (%)		9.6	26.1	13.7	15.5	17.4	16.1	23.8	11.6	9.3	9.3	17.8	20.3	14.1	18.0	10.4	11.9	9.6	
**DALY-Cost**	total DALY (YLL + YLD) *		34.44	5.78	0.28	8.11	53.00	8.31	62.81	39.68	24.24	100.71	56.35	0.37	47.89	43.29	51.26	22.49	0.20	0.0000
	DALY (YLL + YLD)		0.118	0.251	0.003	0.114	0.272	0.025	0.305	0.288	0.094	0.853	0.558	0.003	0.563	0.433	1.068	0.149	0.003	
			1.364	0.811	0.006	0.942	2.062	0.271	2.312	1.700	1.042	3.934	2.714	0.005	2.279	2.557	3.599	0.957	0.005	
	Average cost (excl revalidation) (€) *		8,513	6,699	5,605	7,623	8,194	7,053	8,411	5,201	7,703	7,271	8,120	5,826	6,841	7,423	10,289	6,645	5,270	0.0000
			6,048	7,174	3,308	13,364	11,284	7,015	11,002	6,507	12,707	7,580	7,357	3,605	7,887	6,493	10,177	7,184	3,341	
	Average cost with readmissions (revalidation excl) (€) *		18,065	17,164	22,053	22,782	19,270	19,422	17,357	11,228	15,035	13,832	27,803	10,603	13,668	18,020	13,844	10,934	12,583	0.0000
			11,839	7,128	29,743	33,515	26,978	14,292	16,255	5,763	12,470	8,369	28,358	5,295	17,167	17,282	8,422	5,050	6,874	

*DALYs* Disability-Adjusted Life Years, *YLLs* Years of Life Lost, *YLDs* Years of Life lost due to Disability, *M* Mean, *SD* Standard Deviation

^*^ Kruskal‒Wallis *p* < 0.05 realized only for case mix index, total DALYs, average cost and average cost with readmission

The mean number of DALYs increased (*p* < 0.001) from 0.45 ± 0.76 to 0.63 ± 3.22 for a Charlson index from 0 to 5. It rose gradually (*p* < 0.001) from 0.00 ± 0.00 to 4.98 ± 7.70 for a 1-minor severity index compared to a 4-extreme severity index (Table 4). We measured the same type of change (*p* < 0.001) in mean DALYs (DS) calculated for inpatients with a mortality prediction index from 1-minor (0.01 ± 0.44) to 4-extreme (5.89 ± 7.55) (Table 4).

As shown in Table 4, the average number of DALYs was higher for patients admitted via the emergency department (1.09 ± 3.96; *p* < 0.001), presenting on their own initiative (1.17 ± 4.57; *p* < 0.0001), with hospitalization in intensive care (1.37 ± 4.63; *p* < 0.001), or benefiting from a geriatric liaison (0.61 ± 3.96; *p* < 0.01).

#### Mean DALYs associated with complications

The mean number (SD) of DALYs per patient hospitalized for complications (*n* = 582) was calculated to be 0.96 (3.56), which was significantly higher (*p* < 0.001) than that for conventional hospitalization (Table 4).

The mean DALYs associated with complications were significantly increased for the treatment of pressure ulcers (0.23 ± 1.79 vs. 0.32 ± 0.05; *p* < 0.0001), catheter-related bloodstream infections (1.66 ± 4.05 vs. 0.19 ± 1.67; *p* < 0.001), bleeding and haematomas (0.47 ± 2.59 vs. 0.17 ± 1.52; *p* < 0.001), physiological and/or metabolic disorders (2.95 ± 6.77 vs. 0.16 ± 1.38; *p* < 0.0001), respiratory failure (10.38 ± 9.00 vs. 0.15 ± 1.31; *p* < 0.001), deep vein thrombosis or pulmonary embolism (4.17 ± 8.16 vs. 0.22 ± 1.76; *p* < 0.001), and sepsis (3.93 ± 6.32 vs. 0.21 ± 1.71; *p* < 0.001); the death of a patient most significantly increased the mean DALY (SD) of the stay, which rose from 0.00 (0.02) to 10.53 (6.46) (*p* < 0.001) (Table 4).

### Benchmarking

#### Process and result indicators

In this study, the activity level varied significantly in the 17 hospitals studied: Between 23 cases treated in centre 2 and 336 stays in hospital 6. Five centres (1, 5, 6, 7, and 9) treated more than 50% of the patients (Table 5).

Hospitals 2 (*n* = 23) and 15 (*n* = 48) were the two institutions with the least activity and reported the highest percentage of stays for urgently admitted patients (35.3% and 29.7%) (Table 5).

The mean Charlson index (SD) ranged from 2.0 (1.2) (centre 4) to 3.5 (1.9) (hospital 2) and showed a homogeneous distribution (Table 5).

The mean length (SD) of stay varied from 3.7 (3.9) days for hospital 3 to 17.9 (23.7) days for hospital 2 (centre treating the fewest patients) (Table 2).

Admissions to intensive care differed from one hospital to another: from 0% of stays for hospital 2 to 29% of stays for hospital 14. Two centres (14 and 15) admitted more than 20% of inpatients to the ICU.

In six hospitals (1, 3, 6, 9, 10 and 11), there were fewer than 1% stays in the geriatrics department.

The comparison also showed variability in intrahospital geriatric contact: There was no recourse for hospitals 2, 4, 8, 12, 15, and 17 and for up to 17.4% of patients in centre 5 (Table 5).

The all-complication rate fluctuated from 8.91% (hospital 11) to approximately 40% (hospitals 10 and 15) (Table 5). Finally, readmission rates were high in some hospitals, such as hospitals 2, 7, and 12, which reported 26%, 23%, and 20% of new stays within 30 days of discharge, respectively (Table 5).

Hospitals 11 and 13 were responsible for 20% and 25% of the total mortality, respectively (Table 5).

Table 4 illustrates the DALYs, and hospital costs reported in the 17 hospitals for the management of PAD in 2018. The average DALYs (SD) varied from 0.003 (0.005) in hospitals 12 and 17 to 1.060 (5.599) for hospital centre 15 (Table 5). Hospital 10 had the highest number of DALYs (100.7). Four hospitals (5, 7, 10, and 11) reported more than 272 DALYs, covering 48.8% of inpatients. Hospital 11 had the highest average number of DALYs (YLDs + YLLs) per inpatient and total costs.

The average costs (SD) of a classic hospitalization per patient varied from €5,201 (€6,507) to €10,289 (€10,177), and those of a readmission ranged from €10,683 (€5,295) to €27,803 (€28,358) (Table 5).

#### Data adjustment according to hospital profile

A stepwise linear regression was performed to determine the impact of our predictors (see statistical analyses) on the hospital cost of in-hospital management of peripheral arterial pathology; in our model, a positive and significant relationship was shown (R^2^ = 0.316) (Table 6).

**Table 6:** Result of cost stepwise linear regression

Model Summary						
Model		R	R2	Adjusted R^2^	Standard error of the estimate	
15		0.5630	0.3160	0.3120	0.6148	
Coefficients^a^						
Model		Unstandardized coefficients		Standardized coefficients		
		B	Standard error	Bêta	*t*	*p*
15	(Constant)	8.388	0.052		160.209	0.000
	HCUP code	0.113	0.023	0.093	4.979	0.000
	Age	-0.045	0.012	-0.066	-3.654	0.000
Severity	2—Moderate	0.122	0.030	0.082	4.109	0.000
	3—Major	0.471	0.048	0.206	9.863	0.000
	4—Extreme	0.970	0.096	0.203	10.131	0.000
Charlson index	2	0.115	0.034	0.072	3.377	0.001
	3	0.182	0.039	0.099	4.637	0.000
	4	0.144	0.049	0.060	2.960	0.003
	5	0.179	0.049	0.081	3.679	0.000
Admission type	Emergency via ED	0.256	0.048	0.096	5.353	0.000
Destination	Other hospital	0.335	0.098	0.059	3.421	0.001
	Deceased	-0.206	0.096	-0.040	-2.134	0.033
Unit of care	Intensive care unit	0.773	0.047	0.294	16.556	0.000
	Inpatient geriatric liaison	0.290	0.069	0.074	4.223	0.000
Risk of mortality	2—Moderate	0.071	0.032	0.044	2.247	0.025

^a^Dependent variable: LN_cost

In our model, the independent variables that positively influenced the logarithm of the cost (Table 6) were the HCUP code, age, severity (2-Moderate, 3-Major, 4-Extreme), Charlson index (2, 3, 4, and 5), admissions via the emergency department, transfer to another hospital, death, and admission to intensive care. However, patient death and age showed a significant but negative relationship with the total cost (Table 6).

When the DALY was the dependent variable, the linear regression obtained an R^2^ = 0.579 (Table 7). For DALYs, the variables objectified as significant were the HCUP code, severity (2-moderate, 3-major, 4-extreme), Charlson index 5, admissions via the emergency department, death, referral by another medical specialist, admission to intensive care and a predicted mortality index score of 4-extreme. However, referral by another medical specialist showed a significant but negative relationship with the total DALY (Table 7).

**Table 7:** Result of DALY stepwise linear regression

Model Summary						
Model		R	R2	Adjusted R ^2^	Standard error of the estimate	
10		0.7610	0.5791	0.5774	1.0620	
Coefficients^a^						
Model		Unstandardized coefficients		Standardized coefficients		
		B	Standard error	Bêta	*t*	*p*
10	(Constant)	-6.422	0.053		-121.877	0.000
	HCUP code	0.075	0.038	0.028	1.962	0.050
Severity	2—Moderate	0.260	0.048	0.079	5.405	0.000
	3—Major	0.676	0.079	0.134	8.557	0.000
	4—Extreme	0.651	0.224	0.062	2.905	0.004
Charlson index	5	0.134	0.068	0.028	1.963	0.050
Admission type	Emergency via ED	0.214	0.084	0.036	2.542	0.011
Destination	Decessed	7.273	0.169	0.644	42.995	0.000
Adressage	Consultant	-0.515	0.193	-0.036	-2.663	0.008
Unit of care	Intensive care unit	0.630	0.081	0.109	7.825	0.000
Risk of mortality	4—Extreme	0.593	0.257	0.049	2.311	0.021

^a^Dependent Variable: LN_DALY

Table 7 shows the ratios obtained from our regression model for costs and DALYs. The ratios per hospital were calculated by dividing the mean observed value by the mean value predicted from the regression model. When the ratio was greater than 1, the observed value was greater than the value predicted by our model.

**Fig. 1:**
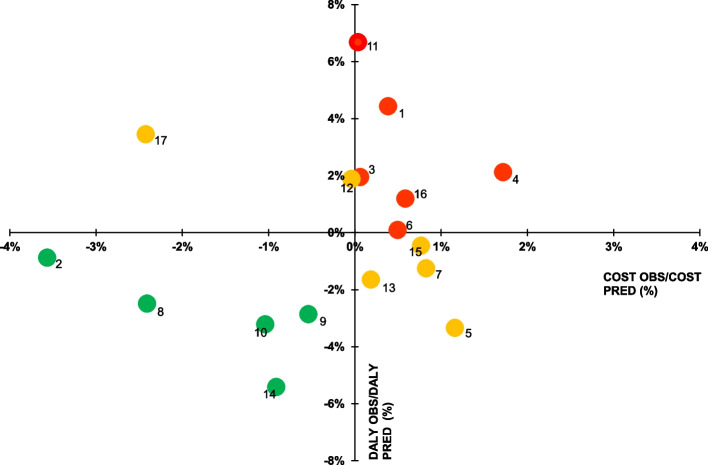
Graphic of observed cost/predicted cost vs. observed DALY/predicted DALY from linear regression among 17 Belgian hospitals. This graph identifies the position of hospitals regarding their performance in terms of costs and DALYs. A bubble represents a hospital and is based on the data from Table 6. Hospitals with a red colour suggest that both variables are unfavourable, hospitals with a green colour suggest that both variables are favourable and hospitals with an orange colour suggest that one of the two variables of the hospital is favourable. DALY, disability-adjusted life year

To identify hospitals more easily with higher observed ratios than predicted, we have translated these data into a four-zone graph. The upper right area shows hospitals with higher than predicted costs and DALYs. The bottom left area depicts hospitals with costs and DALYs lower than the calculated forecast (Fig. 1).

Hospitals 1, 3, 4, 6, 11, and 16 seemed to have higher costs and DALYs than the values ​​predicted in our model (Table 8 and Fig. 1). The difference between the average cost observed and the average cost predicted for hospital 4 was €534 for a normal stay and €1,595 for a readmission. With 71 stays including 11 readmissions, the additional hospital cost therefore amounted to €49,585. We observed lower values ​​than those calculated in our model for costs and DALYs in hospitals 2, 8, 9, and 10 alone. The costs observed for hospitals 12 and 17 were lower than those in our model. Hospitals 5, 7, 13, and 15 reported lower DALYs than those calculated in our model. The total observed costs were 2% lower than the predicted total costs.

**Table 8:** Results of observed cost/predicted cost and observed DALYs/predicted DALYs from linear regression for 17 hospitals

Hospitals	Observed/predicted cost	Observed/predicted DALYs
1	1.004	0.961
2	0.964	0.973
3	1.001	0.981
4	1.017	0.996
5	1.012	1.047
6	1.005	1.004
7	1.008	1.021
8	0.976	1.001
9	0.995	1.024
10	0.990	1.023
11	1.000	0.938
12	1.000	0.981
13	1.002	1.019
14	0.991	1.048
15	1.008	1.012
16	1.006	0.994
17	0.976	0.943

*DALYs* Disability-adjusted life years

## Discussion

The objective of our study was to carry out an evaluation-comparison of costs-DALY in the management of PAD in 17 Belgian general and university hospitals. To assess facilities, we identified hospital costs as well as the impact of DALYs on managing PAD in hospitals.

PAD is the third leading cause of atherosclerotic vascular disease. Globally, in 2010, more than 200 million people suffered from this condition, but its incidence has increased by 23.5% over the past ten years [24]. In a study using the US Administrative Database, the prevalence of PAD ranged from 3 to 4% in middle-aged adults and from 13 to 14% in older adults [25]. Disability and mortality associated with PAD have increased significantly over the past twenty years [4]. There is also evidence that the economic costs of hospitalization for PAD have become equivalent to or even higher than those for coronary heart disease or cerebrovascular disease, which require major cardiovascular interventions [26].

In the absence of publication of good practice recommendations in Belgium, we relied on the literature and the availability of medico-administrative data to identify specific indicators of PAD.

The male (M)/female (F) ratio in the patients included in our study was M-67%/F-33% and comparable to that reported in the literature [27–29]. Numerous clinical studies demonstrate strong associations between sex, severity, and progression of PAD. Contrary to popular belief, PAD is not significantly more common in men when defined by the arm-ankle index [2]. However, patients are less likely to receive preventive statin therapy as recommended [30] and are more likely to present at older ages with a more severe clinical diagnosis of chronic limb-threatening ischaemia [31]. Women with PAD have a higher risk profile and resource use [32], as evidenced by a higher rate of urgent hospitalization [33], longer in-hospital length of stay [32], higher rates of short-term postoperative complications [34, 35] and higher mortality [36]. Finally, female patients with symptomatic PAD tend to report greater disability than males [37].

The mean age (SD) of patients in our population was 68.5 (10.9 years), which was comparable to recently published values [29, 38]. Authors have shown the impact of patients’ functional status on the results of lower limb revascularization for critical ischaemia in the elderly [39]: dependent patients were older than nondependent patients and had a more extensive cardiovascular and metabolic history, resulting in morbidity, 30-day mortality and a significantly higher cost for their management [39].

Until now, advances in vascular surgery and geriatrics have developed largely independently of each other, and there is – unlike orthogeriatric medicine – virtually no overlap in daily clinical practice. However, in an interdisciplinary setting, geriatric concepts could be helpful in therapeutic decision-making and prognosis in vascular surgery, especially for patients older than 85 years [40, 41]. Although geriatric patients represented only 6% of the total stays in our study, the admission of a patient to a geriatric unit reduced the average cost (SD) of care from €8,571 (€8,691) to €2,383 (€2,437), while that of hospitalization in vascular surgery with a geriatric connection was €13,551 (€12,008). It should be noted, however, that a study in twenty-one regions around the world showed that the burden of PAD was no longer limited to the elderly population but now affected young adults; this observation will inevitably have significant financial repercussions [4].

In our work, we calculated that, depending on the hospital, the average cost (DS) of a classic hospitalization varied from €5,201 (€6,507) to €10,289 (€10,177) and that of a readmission fluctuated from €10,683 (€5,295) to €27,803 (€28,358); these amounts seem consistent with the reported figures [38, 42–44]. Nevertheless, a review of the literature reveals significant differences in the costs calculated for the management of peripheral arterial pathologies around the world [45]. These differences may be partly explained by the methodology of the studies and partly by the models of organization of care in the different countries [38, 42–44].

Moreover, for a decade, the costs related to the management of peripheral arterial disease have significantly increased and have been largely influenced by the growing global number of patients with PAD, the degree of severity of the vascular disease and the endovascular or surgical techniques used [46]. Thus, in a recent Australian study, the average cost per admission for the endovascular revascularization group was AUD$18,396 (= €12,038), that of open surgery AUD$31,908 (= €20,880) and that of the amputation group adults AUD$43,033 (= €28,161) [43].

High costs could quickly become a factor limiting access to adequate care, as suggested by a study showing that most patients with critical limb ischaemia are no longer financially adequately covered by current Medicare reimbursement after open bypass surgery [38].

As we have shown in a previous study, the weighting of complications and the adjustment of the results to the case mix index are essential for discriminating between hospitals [11, 47]. The calculation of DALYs considers the YLLs through mortality and disability because of medical complications on the daily life of patients. For this reason, in-hospital age-specific mortality is the most important indicator, particularly when a young patient is lost. Here, we measured a mortality rate of 2% in the 17 hospitals studied, equivalent to the measurements reported in the relevant literature [48].

The cost-DALY impact of readmissions is not negligible for some hospitals and should be considered when evaluating hospital performance. The average duration (SD) of stay was 6.6 days (11.4 days), which was comparable [49] to or lower than the values reported in the literature, namely, 11.1 days (10.1 days) [46] or 15.7 days (12 days) [29]. Nevertheless, we must point out that our figures are probably slightly underestimated since they cover both surgical (86%) and medical (14%) care and exclude data related to rehabilitation care. In this study, we did not analyse the data from the rehabilitation services to allow a reliable comparison given that this type of service is not found in all the benchmarked hospitals.

In patients undergoing lower extremity bypass surgery, length of stay is primarily associated with the occurrence of postoperative complications [50], while readmission at 30 days is largely explained by the patient’s underlying disease [51–54]. In addition, prolonging the length of stay is an independent risk factor for readmissions. Our results therefore suggest that efforts to reduce both length of stay and readmission will be complementary.

In our study, the readmission rate was 14%, comparable to that in the literature [52]. Gonzalez and colleagues [53] showed that readmission rates varied considerably depending on the indication: 7.3% (intermittent claudication) versus 19.5% (high risk). However, the literature suggests adjusting the length of stay and 30-day readmission rates based on risk [51]. To this end, our study demonstrated that even after adjusting for patient demographics, length of stay, and discharge destination, high-risk patients were significantly more likely to be readmitted. We also showed that the proportion of high-risk patients identified by the Charlson index was related to the readmission rate by institution (R^2^ = 0.3106; *p* < 0.05).

The DALYs therefore allowed us to weight the complications encountered during the stays, considering that they do not all have the same impact on the patient’s outcome. To develop the patient safety indicators, we used the construction methodology of the AHRQ, V.5.0 [14], which does not include dehiscence and infection of operative wounds in vascular surgery. The objectified complication rate in the 17 hospitals was 24% and within the range of the figures presented in the literature [42]. Among these complications, surgical site infection is reported as the most common cause of readmission, followed by bleeding/haemorrhage or haematoma [52]. In terms of outcome indicators, bleeding/haemorrhages or haematomas had a higher frequency in our study (20%) than in the recent literature (5.8% to 6.8%) [54]; this observation can potentially be explained by the systematic reporting of postoperative haematoma described in the letters of stay or the operating protocols. Belgian funding rules may indeed encourage some hospitals to over- or underscore certain information to optimize their funding [55]. The DALY weighting used for this complication moderates the magnitude of its frequency.

Our article therefore highlights the interest of combining costs and quality indicators to assess the performance of hospitals. The weighting of medical complications and the adjustment of our analysis according to the hospital case mix index illustrated the waste encountered to optimize the wealth available. Policy makers should reward the appropriateness of care rather than the amount of treatment provided.

The literature reports a significant improvement in the results of patient care if the principle of evidence-based medicine is introduced and applied during care for a pathology [7, 8, 56–58], particularly when provider comparison of structural, process, and outcome indicators is used to improve team performance and patient outcome [7, 8]. However, this approach also requires the involvement of the patient through the evaluation of their satisfaction. A recently published review focused on patient-reported outcome measures (PROMS) for arterial vascular surgery [59]. The authors identified a lack of awareness of existing PROMS, knowledge of how PROMS are developed and validated, and clarity on how PROMS should be used by the clinician in the field [59].

Furthermore, being treated for PAD in a certified hospital and by a team with significant exposure to the pathology provides better results at lower costs. This may be due to the greater use of treatments aimed at preserving limb function [60, 61]. The two hospitals with the smallest number of patients had the longest length of stay, treated the most emergency patients, did not use liaison geriatrics, and had a higher number of readmissions.

In the absence of a systematic comparative analysis, such as benchmarking, Belgian hospital managers and healthcare providers are not able to assess the quality and efficiency of their procedures in the overall management of a particular pathology. For this reason, our study proposes a benchmark that reflects the organization of hospitals without any application of guidelines and before weighting the results of the care provided [62, 63]. We believe that the use of the DALY is a good approach to evaluate the results of care pathways in hospitals, translating adverse events into a valuable common unit of comparison in the field of care quality management.

Combined with PROMS, the automatic availability of our methodology in the daily life of healthcare actors could be a concrete approach to translate "the added value" brought to patients and society for each care approach.

Our multidimensional reporting (regression, Donabedien model) first identifies the best performing hospitals in terms of cost and patient safety. It then gives actors in the field the opportunity to identify the elements of the most impactful care processes, compared to other establishments, for the same pathology.

After having identified and prioritized the indicators that justify the position of their institutions in relation to others, the managers of the establishments can then mobilize the most appropriate actors (managers, doctors, nurses, pharmacists, financial analysts, etc.) to set objectives consistent during medico-economic meetings and activate the levers of positive change in hospitals.

Finally, due to the limited availability of financial resources, prevention should be a primary goal in the management of PAD. The assessment of overall cardiovascular risk and the presence of related factors are the foundations of preventive vascular medicine. However, educational factors such as dietary habits and physical activity levels must also be considered. For secondary prevention, people at very high risk should be more systematically identified to benefit from more intensive medical treatment.

This approach must therefore be a prioritized societal and individual objective. Nevertheless, risk assessment remains an inexact science, and although it may be useful for assessing risk in populations of subjects, application to the individual patient is still too limited. However, new emerging methods for integrating sociodemographic, genetic, clinical, and lifestyle measures will hopefully improve the accuracy of risk prediction for each patient [64].

This study has some limitations. The impact of DALYs was probably greater than what we estimated in our study. Indeed, the scope of the study was limited to the hospital environment, neglecting all the complications treated in consultation or by other nonhospital care providers. Furthermore, it seems essential to refine the key weightings to improve the quality of the comparisons. As we only had data from 2018 from hospitals participating in the PACHA project, we were unable to identify the following readmissions:

- those occurring within 30 days for stays where the person was admitted to hospital after December 2, 2018.
- hospitalized patients who may have been admitted to a different establishment at the beginning.

Unfortunately, neither the ICD-10-PCS coding system nor the Belgian nomenclature fully distinguished the surgical approaches used in this study.

## Conclusions

Assessing the value of patient safety indicators associated with costs is a prerequisite for quality-improvement and financial-efficiency efforts made by managers and practitioners.

However, access to benchmarking to assess the costs of hospital care must be refined and integrated into the steps put in place to improve the quality of care provided by hospitals. This appears to be essential for evaluating all care pathways using a comparable unit of measurement.

The case mix index of the hospital must also be considered in the comparative analysis at the risk of drawing erroneous conclusions.

Finally, other indicators should be added to our study, including patient evaluation of the results obtained (PROMS).

The education of practitioners and other hospital and extrahospital actors will be a crucial step in the deployment of the method.

Faced with the growing demand in the field of medico-economic tools, we believe that our approach may open a new door facilitating different management of available resources.

Population increase and ageing will sooner or later impose this type of approach for the proper use of common resources to guarantee the provision of medical care of equivalent quality to all patients.

## Data Availability

Data may be obtained from a third party and are not publicly available. The data sets generated and/or analysed during the current study are not publicly available, as this study is partly based on cost data from a hospital’s benchmarking of cost by pathology. They are available from the corresponding author upon reasonable request and after being rendered anonymous. (Benoit RONDELET, Direction Médicale, CHU UCL Namur, Avenue G. Therasse, 1, 5530 – Yvoir, Belgium—E-mail: benoit.rondelet@chuuclnamur.uclouvain.be).

## References

[1] Lu Y, Cui X, Zhang L, Wang X, Xu Y, Qin Z, Liu G, Wang Q, Tian K, Lim KS, Charles CJ, Zhang J, Tang J (2022). The functional role of lipoproteins in atherosclerosis: novel directions for diagnosis and targeting therapy. Aging Dis..

[2] Criqui MH, Aboyans V (2015). Epidemiology of peripheral artery disease. Circ Res.

[3] WHO Study Group on the Classification of Atherosclerotic Lesions, World Health Organization: Classification of Atherosclerotic Lesions: Report of a Study Group [Met in Washington, October 7–11, 1957]. Technical Report Series; No. 143. Geneva: World Health Organization. 1958 13558199

[4] Sampson UK, Fowkes FG, McDermott MM, Criqui MH, Aboyans V, Norman PE, Forouzanfar MH, Naghavi M, Song Y, Harrell FE, Denenberg JO, Mensah GA, Ezzati M, Murray C (2014). Global and regional burden of death and disability from peripheral artery disease: 21 world regions, 1990 to 2010. Glob Heart.

[5] Riviere AB, Bouée S, Laurendeau C, Torreton E, Gourmelen J, Thomas-Delecourt F (2018). Outcomes and management costs of peripheral arterial disease in France. J Vasc Surg.

[6] de Mestral C, Salata K, Hussain MA, Kayssi A, Al-Omran M, Roche-Nagle G (2019). Evaluating quality metrics and cost after discharge: a population-based cohort study of value in health care following elective major vascular surgery. Ann Surg.

[7] Abramson BL, Al-Omran M, Anand SS, Albalawi Z, Coutinho T, de Mestral C, Dubois L, Gill HL, Greco E, Guzman R, Herman C, Hussain MA, Huckell VF, Jetty P, Kaplovitch E, Karlstedt E, Kayssi A, Lindsay T, Mancini GBJ, McClure G, McMurtry MS, Mir H, Nagpal S, Nault P, Nguyen T, Petrasek P, Rannelli L, Roberts DJ, Roussin A, Saw J, Srivaratharajah K, Stone J, Szalay D, Wan D, Cox H, Verma S, Virani S (2022). Canadian cardiovascular society 2022 guidelines for peripheral arterial disease. Can J Cardiol.

[8] Benson RA, Okoth K, Keerthy D, Gokhale K, Adderley NJ, Nirantharakumar K, Lasserson DS (2022). Analysis of the relationship between sex and prescriptions for guideline-recommended therapy in peripheral arterial disease, in relation to 1-year all-cause mortality: a primary care cohort study. BMJ Open.

[9] Slawomirski L, Auraaen A, Klazinga N. The economics of patient safety. Strengthening a value-based approach to reducing patient harm at national level, OECD health working paper. 96, 20. http://www.oecd.org/health/patient-safety.htm. 2017.

[10] Pirson M, Leclercq P (2014). Un projet pilote d’évaluation des coûts par pathologie, le projet PACHA. Healthc Exec.

[11] Dehanne F, Gourdin M, Devleesschauwer B, Bihin B, Van Wilder P, Mareschal B, Leclercq P, Pirson M (2021). Cost-DALY comparison of hip replacement care in 12 Belgian hospitals. BMJ Open Qual.

[12] Spf Santé Pulique: Sécurité de la chaîne alimentaire et environnement. Liste des codes ICD-10-BE. https://www.health.belgium.be/fr/sante/organisation-des-soins-de-sante/hopitaux/systemes-denregistrement/icd-10-be/publications#reflist

[13] The Agency for Healthcare Research and Quality (AHRQ), The Healthcare Cost and Utilization Project (HCUP): Clinical Classifications Software (CCS) for ICD-10-PCS (beta version). https://www.hcup-us.ahrq.gov/toolssoftware/ccs10/ccs10.jsp

[14] The Agency for Healthcare Research and Quality (AHRQ): Toolkit for using the AHRQ quality indicators: fact sheet on patient safety indicators. https://www.ahrq.gov/sites/default/files/wysiwyg/professionals/systems/hospital/qitoolkit/combined/a1b_combo_psifactsheet.pdf

[15] Quan H, Sundararajan V, Halfon P, Fong A, Burnand B, Luthi JC, Saunders LD, Beck CA, Feasby TE, Ghali WA (2005). Coding algorithms for defining comorbidities in ICD-9-CM and ICD-10 administrative data. Med Care.

[16] Health Statistics and Information Systems: Metrics: disability-adjusted life year (DALY). https://www.who.int/healthinfo/global_burden_disease/metrics_daly/en/ (2018)

[17] Institute for Health Metrics and Evaluation: Global burden of disease study 2016, data resources. http://ghdx.healthdata.org/gbd-2016 (2018)

[18] Jha AK, Larizgoitia I, Audera-Lopez C, Prasopa-Plaizier N, Waters H, Bates DW (2013). The global burden of unsafe medical care: analytic modelling of observational studies. BMJ Qual Saf.

[19] Statbel Data.gov.be.: Tables de mortalité et espérance de vie 2021. https://data.gov.be/fr/dataset/72c1db031defb669a78ea81ddba786bc3238a78a

[20] Jones DW, Farber A (2020). Review of the global vascular guidelines on the management of chronic limb-threatening Ischemia. JAMA Surg.

[21] Vemulapalli S, Patel MR, Jones WS (2015). Limb Ischemia: cardiovascular diagnosis and management from head to toe. Curr Cardiol Rep.

[22] Touma J, Tacher V, Cochennec F, Kobeiter H, Raux M, Allaire E, Marzelle J. Chirurgie Endovasculaire Aorto-Iliaque et des Membres Inférieurs Pour Pathologie Occlusive Athéromateuse [43–029-J]. EMC, Techniques Chirurgicales – Chirurgie Vasculaire. 2017

[23] Ricco JB, Belmonte R, Schneider F. Chirurgie des Artères du Membre Inférieur: Complications [43–029-H]. EMC, Techniques Chirurgicales – Chirurgie Vasculaire (2016)

[24] Fowkes FG, Rudan D, Rudan I, Aboyans V, Denenberg JO, McDermott MM, Norman PE, Sampson UK, Williams LJ, Mensah GA, Criqui MH (2013). Comparison of global estimates of prevalence and risk factors for peripheral artery disease in 2000 and 2010: a systematic review and analysis. Lancet.

[25] Selvin E, Erlinger TP (2004). Prevalence of and risk factors for peripheral arterial disease in the United States: results from the National Health and nutrition examination survey, 1999–2000. Circulation.

[26] Smolderen KG, Wang K, de Pouvourville G, Brüggenjürgen B, Röther J, Zeymer U, Parhofer KG, Steg PG, Bhatt DL, Magnuson EA (2012). Two-year vascular hospitalisation rates and associated costs in patients at risk of atherothrombosis in France and Germany: highest burden for peripheral arterial disease. Eur J Vasc Endovasc Surg.

[27] Aziz F, Lehman E, Blebea J, Lurie F (2018). Postoperative complications after lower extremity arterial bypass increase the risk of new deep venous thrombosis. Phlebology.

[28] Jain AK, Kalbaugh CA, Farber MA, Marston WA, Vallabhaneni R (2014). Race and gender affect outcomes of lower extremity bypass. J Vasc Surg.

[29] Wang GJ, Jackson BM, Foley PJ, Damrauer SM, Kalapatapu V, Golden MA, Fairman RM (2017). Treating peripheral artery disease in the wake of rising costs and protracted length of stay. Ann Vasc Surg.

[30] McGinigle KL, Browder SE, Strassle PD, Shalhub S, Harris LM, Minc SD (2021). Sex-related disparities in intervention rates and type of intervention in patients with aortic and peripheral arterial diseases in the National Inpatient Sample Database. J Vasc Surg.

[31] Jackson EA, Munir K, Schreiber T, Rubin JR, Cuff R, Gallagher KA, Henke PK, Gurm HS, Grossman PM (2014). Impact of sex on morbidity and mortality rates after lower extremity interventions for peripheral arterial disease: observations from the blue cross blue shield of Michigan cardiovascular consortium. J Am Coll Cardiol.

[32] Doshi R, Patel K, Desai R, Patel P, Grines C, Meraj P (2020). Differences in risk factors and resource utilization for women undergoing percutaneous coronary intervention and lower extremity peripheral vascular intervention. Catheter Cardiovasc Interv.

[33] Egorova N, Vouyouka AG, Quin J, Guillerme S, Moskowitz A, Marin M, Faries PL (2010). Analysis of gender-related differences in lower extremity peripheral arterial disease. J Vasc Surg..

[34] Mays BW, Towne JB, Fitzpatrick CM, Smart SC, Cambria RA, Seabrook GR, Freischlag JA (1999). Women have increased risk of perioperative myocardial infarction and higher long-term mortality rates after lower extremity arterial bypass grafting. J Vasc Surg..

[35] Jain AK, Velazquez-Ramirez G, Goodney PP, Edwards MS, Corriere MA (2011). Gender-based analysis of perioperative outcomes associated with lower extremity bypass. Am Surg.

[36] Elbadawi A, Barssoum K, Megaly M, Rai D, Elsherbeeny A, Mansoor H, Shishehbor MH, Abdel-Latif A, Gulati M, Elgendy IY (2021). Sex differences in trends and in-hospital outcomes among patients with critical limb Ischemia: a nationwide analysis. J Am Heart Assoc.

[37] Enzler MA, Ruoss M, Seifert B, Berger M (1996). The influence of gender on the outcome of arterial procedures in the lower extremity. Eur J Vasc Endovasc Surg.

[38] Voicu S, Trooboff SW, Goodney PP, Zwolak RM, Powell RJ (2020). Medicare reimbursement of lower extremity bypass does not cover cost of care for most patients with critical limb Ischemia. J Vasc Surg.

[39] Madou ID, Slade MD, Orion KC, Sarac T, Chaar CIO (2017). The impact of functional status on the outcomes of endovascular lower extremity revascularization for critical limb Ischemia in the elderly. Ann Vasc Surg.

[40] Meulenbroek AL, van Mil SR, Faes MC, Mattace-Raso FUS, Fourneau I, van der Laan L (2022). A systematic review of strategies for preventing delirium in patients undergoing vascular surgery. Ann Vasc Surg.

[41] Maassen B, Chondros K, Bollheimer LC (2021). Innovativ: geriatrische Konzepte für die Gefäßmedizin und Gefäßchirurgie. Gefässchirurgie.

[42] Tsay C, Luo J, Zhang Y, Attaran R, Dardik A, Chaar CIO (2020). Perioperative outcomes of lower extremity revascularization for rest pain and tissue loss. Ann Vasc Surg.

[43] Tang L, Paravastu SCV, Thomas SD, Tan E, Farmer E, Varcoe RL (2018). Cost analysis of initial treatment with endovascular revascularization, open surgery, or primary major amputation in patients with peripheral artery disease. J Endovasc Ther.

[44] Kohn CG, Alberts MJ, Peacock WF, Bunz TJ, Coleman CI (2019). Cost and inpatient burden of peripheral artery disease: findings from the National Inpatient Sample. Atherosclerosis.

[45] Behrendt CA, Sigvant B, Kuchenbecker J, Grima MJ, Schermerhorn M, Thomson IA, Altreuther M, Setacci C, Svetlikov A, Laxdal EH, Goncalves FB, Secemsky EA, Debus ES, Cassar K, Beiles B, Beck AW, Mani K, Bertges D (2020). Editor’s choice - international variations and sex disparities in the treatment of peripheral arterial occlusive disease: a report from VASCUNET and the international consortium of vascular registries. Eur J Vasc Endovasc Surg.

[46] Seo A, Yamamoto K, Akai A, Akagi D, Takayama T, Hoshina K (2018). The relationship between medical expenses and the severity of peripheral arterial disease in Japan. Heart Vessels.

[47] Pirson M, Dehanne F, Van den Bulcke J, Leclercq P, Martins D, De Wever A (2018). Evaluation of cost and length of stay, linked to complications associated with major surgical procedures. Acta Clin Belg.

[48] Nakazawa KR, Cornwall JW, Rao A, Han DK, Ting W, Tadros RO, Faries PL, Vouyouka AG (2021). Trends, factors, and disparities associated with length of stay after lower extremity bypass for tissue loss. J Vasc Surg.

[49] Siracuse JJ, Gill HL, Jones DW, Schneider DB, Connolly PH, Parrack I, Huang ZS, Meltzer AJ (2014). Risk factors for protracted postoperative length of stay after lower extremity bypass for critical limb Ischemia. Ann Vasc Surg.

[50] Witcher A, Axley J, Novak Z, Laygo-Prickett M, Guthrie M, Xhaja A, Chu DI, Brokus SD, Spangler EL, Passman MA, McGinigle KL, Pearce BJ, Schlitz R, Short RT, Simmons JW, Cross RC, McFarland GE, Beck AW (2021). Implementation of an enhanced recovery program for lower extremity bypass. J Vasc Surg.

[51] Damrauer SM, Gaffey AC, DeBord Smith A, Fairman RM, Nguyen LL (2015). Comparison of risk factors for length of stay and readmission following lower extremity bypass surgery. J Vasc Surg.

[52] Syed MH, Hussain MA, Khoshhal Z, Salata K, Altuwaijri B, Hughes B, Alsaif N, de Mestral C, Verma S, Al-Omran M (2018). Thirty-day hospital readmission and emergency department visits after vascular surgery: a Canadian prospective cohort study. Can J Surg.

[53] Gonzalez AA, Cruz CG, Dev S, Osborne NH (2016). Indication for lower extremity revascularization and hospital profiling of readmissions. Ann Vasc Surg.

[54] Zghouzi M, Moussa Pacha H, Ullah W, Sattar Y, Ahmad B, Osman H, Mohamed MO, Mir T, Banerjee S, Shishehbor MH, Prasad A, Rits Y, Mamas MA, Alraies MC (2021). In-hospital outcomes of endovascular versus surgical revascularization for chronic total occlusion in peripheral artery disease. Catheter Cardiovasc Interv.

[55] Service public fédéral, Santé Publique, sécurité de la chaine alimentaire et environnement, Financement des hôpitaux – Réforme du paysage hospitalier et du financement des hôpitaux. https://www.health.belgium.be/fr/sante/organisation-dessoins-desante/hopitaux/financement-des-hopitaux/reforme-dupaysage (2018)

[56] Magnowski A, Lindquist JD, Herzog EC, Jensen A, Dybul SL, Trivedi PS (2022). Changes in the national endovascular management of femoropopliteal arterial disease: an analysis of the 2011–2019 medicare data. J Vasc Interv Radiol.

[57] Lazzarini PA, Raspovic A, Prentice J, Commons RJ, Fitridge RA, Charles J, Cheney J, Purcell N, Twigg SM (2022). Guidelines development protocol and findings: part of the 2021 Australian evidence-based guidelines for diabetes-related foot disease. J Foot Ankle Res.

[58] Wong ND, Budoff MJ, Ferdinand K, Graham IM, Michos ED, Reddy T, Shapiro MD, Toth PP (2022). Atherosclerotic cardiovascular disease risk assessment: an American Society for Preventive Cardiology clinical practice statement. Am J Prev Cardiol.

[59] Hicks CW, Vavra AK, Goldsborough E, Rebuffatti M, Almeida J, Duwayri YM, Haurani M, Ross CB, Shah SK, Shireman PK, Smolock CJ, Yi J, Woo K (2021). Current status of patient-reported outcome measures in vascular surgery. J Vasc Surg.

[60] Decker JA, Schwarz F, Kroencke TJ, Scheurig-Muenkler C. The in-hospital care of patients with peripheral arterial occlusive disease–the effects of hospital size and certification status. Dtsch Arztebl Int Arztebl.m2022.0235 (2022). 10.3238/arztebl.m2022.0235 10.3238/arztebl.m2022.0235 PMC9756319 35734915

[61] Elbadawi A, Elgendy IY, Rai D, Mahtta D, Megaly M, Pershad A, Denktas A, Brilakis ES, Drachman DE, Banerjee S, Shishehbor MH, Jneid H (2021). Impact of hospital procedural volume on outcomes after endovascular revascularization for critical limb Ischemia. JACC Cardiovasc Interv.

[62] Service public fédéral, Santé Publique, sécurité de la chaine alimentaire et environnement. Programme Pay for Performance 2018 pour les hôpitaux généraux, note d’accompagnement. https://www.health.belgium.be/sites/default/files/uploads/fields/fpsheal (2019)

[63] Annemans L, Boeckxstaens P, Borgermans L. Avantages, Désavantages et Faisabilité De L’introduction De Programmes “P4Q” en Belgique. Centre Fédéral D’expertise des Soins de Santé, Brussels, Belgium (2009)

[64] Foryciarz A, Pfohl SR, Patel B, Shah N (2022). Evaluating algorithmic fairness in the presence of clinical guidelines: the case of atherosclerotic cardiovascular disease risk estimation. BMJ Health Care Inform.

